# Death of Dr. Orrin Smith

**Published:** 1867-10

**Authors:** 


### *Death of Dr. Orrin Smith.*

RESOLUTIONS OF THE CHICAGO MEDICAL SOCIETY.—At the last meeting of the Chicago Medical Society, the following resolutions were adopted, in view of the recent death of Dr. Smith:

*Whereas*, It has pleased the Supreme Ruler to remove from our midst and take to Himself a prominent member of this Society, by the death of Dr. Orrin Smith; therefore, as a tribute of respect, we offer the following resolutions:

*Resolved*, That. in the death of Dr. Smith, this Society has been deprived of an honorable member, the community of an experienced practitioner and a valued citizen.

*Resolved*, That we recognize in our departed friend and brother a man of great energy and perseverance, so entirely devoted to his profession and to the cause of humanity that, although advanced in life, and for many years a sufferer with organic disease of the heart, he continued to practice his profession till a few weeks of his death.

*Resolved*, That we tender to the afflicted family of the deceased our earnest sympathy in this their sad bereavement and irreparable loss.

*Resolved*, That the Secretary of this Society furnish a copy of these resolutions to the family of the deceased, to each of the daily papers of the city, and to the Chicago *Medical Journal* and *Medical Examiner*.

MODERN ANÆSTHESIA. By the Hon. Truman Smith. A republication of the evidences that Horace Wells, evidently a high minded and usitive gentleman, discovered the principle and method of Anæsthesia. Our own memory, contemporaneous with the facts, convinces us that a man named Morton, with a large proportion of the alphabet prefixed to his name, was the real father of both. But God preserve us from his gratitude for expressing our belief! Our pockets ache at the suggestion.

HEADLAND ON THE ACTION OF MEDICINES. This fifth "revised and enlarged edition," is worthy of its already acheived popularity. Every intelligent physician is familiar (or should be) with its merits.

